# Supplementary material for: Higher eQTL power reveals signals that boost GWAS colocalization
Source: bioRxiv. 2025 Aug 5:2025.08.05.668745. Preprint. [Version 1] doi: 10.1101/2025.08.05.668745 (PMC12340864; doi:10.1101/2025.08.05.668745)
Supplement: Supplement 1 [file NIHPP2025.08.05.668745v1-supplement-1.pdf]

bioRxiv preprint doi: https://doi.org/10.1101/2025.08.05.668745; this version posted August 5, 2025. The copyright holder for this preprint (which was not certified by peer review) is the author/funder, who has granted bioRxiv a license to display the preprint in perpetuity. It is made available under aCC-BY 4.0 International license.

Supplemental Figures

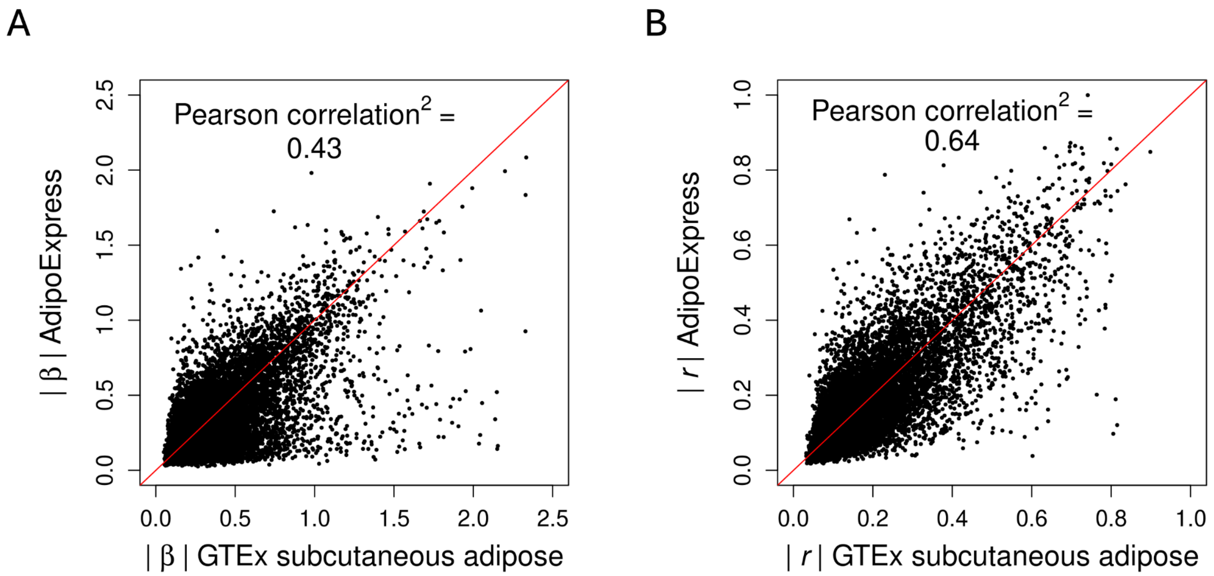

Figure S1. Concordance of effect sizes between AdipoExpress and GTEx subcutaneous adipose. A) Comparison of the absolute values of the (A) beta coefficients and (B) r value for primary eQTL signals for overlapping genes between GTEx v10 subcutaneous adipose (x-axis) and AdipoExpress (y-axis).

bioRxiv preprint doi: https://doi.org/10.1101/2025.08.05.668745; this version posted August 5, 2025. The copyright holder for this preprint (which was not certified by peer review) is the author/funder, who has granted bioRxiv a license to display the preprint in perpetuity. It is made available under aCC-BY 4.0 International license.

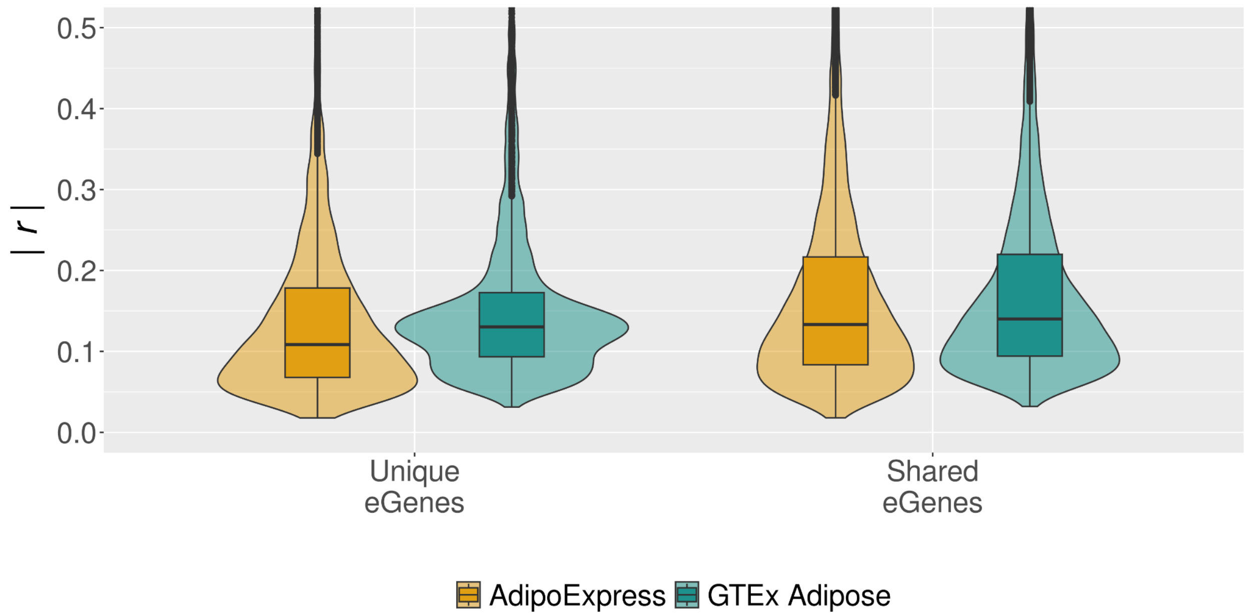

Figure S2. Comparison of effect size between AdipoExpress and GTEx subcutaneous adipose. Absolute value of *r* (y-axis) for primary signals in the set of overlapping eGenes (right) and non-overlapping genes (left) for both AdipoExpress and GTEx v10 subcutaneous adipose. The top, middle, and bottom of the boxplot inside each violin represents the 75th, 50th and 25th percentiles of the values, respectively.

bioRxiv preprint doi: https://doi.org/10.1101/2025.08.05.668745; this version posted August 5, 2025. The copyright holder for this preprint (which was not certified by peer review) is the author/funder, who has granted bioRxiv a license to display the preprint in perpetuity. It is made available under aCC-BY 4.0 International license.

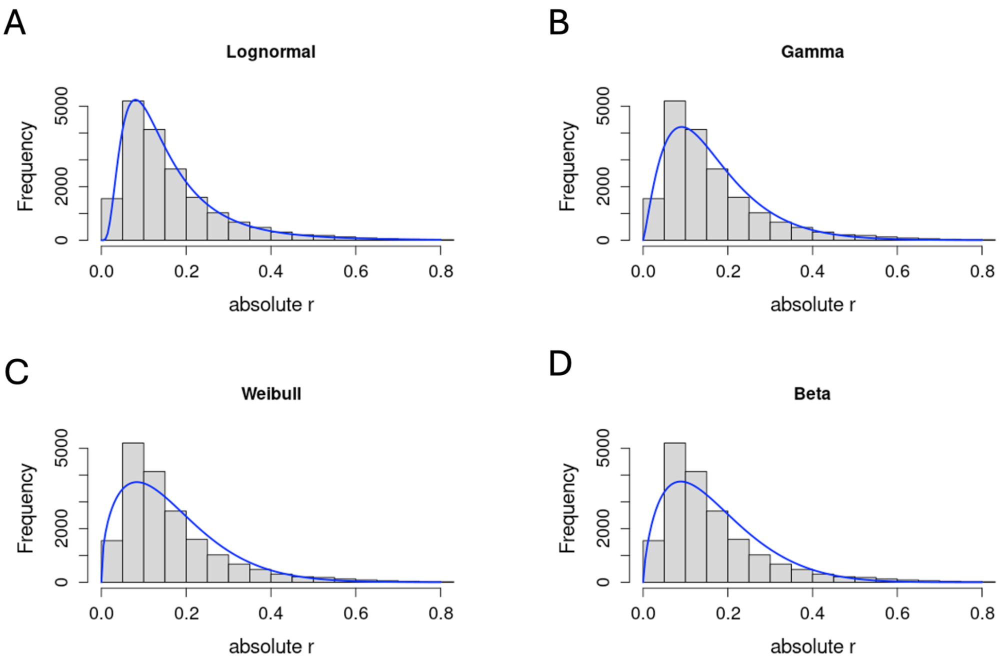

Figure S3. Fitting parametric distributions to an empirical distribution of eQTL effect sizes. A-D) Histograms of observed absolute r for primary signals in AdipoExpress with fitted distributions overlaid (blue lines). Distributions are lognormal (A), gamma (B), Weibull (C), and beta (D) and were fitted using the "fit" function in R.

bioRxiv preprint doi: https://doi.org/10.1101/2025.08.05.668745; this version posted August 5, 2025. The copyright holder for this preprint (which was not certified by peer review) is the author/funder, who has granted bioRxiv a license to display the preprint in perpetuity. It is made available under aCC-BY 4.0 International license.

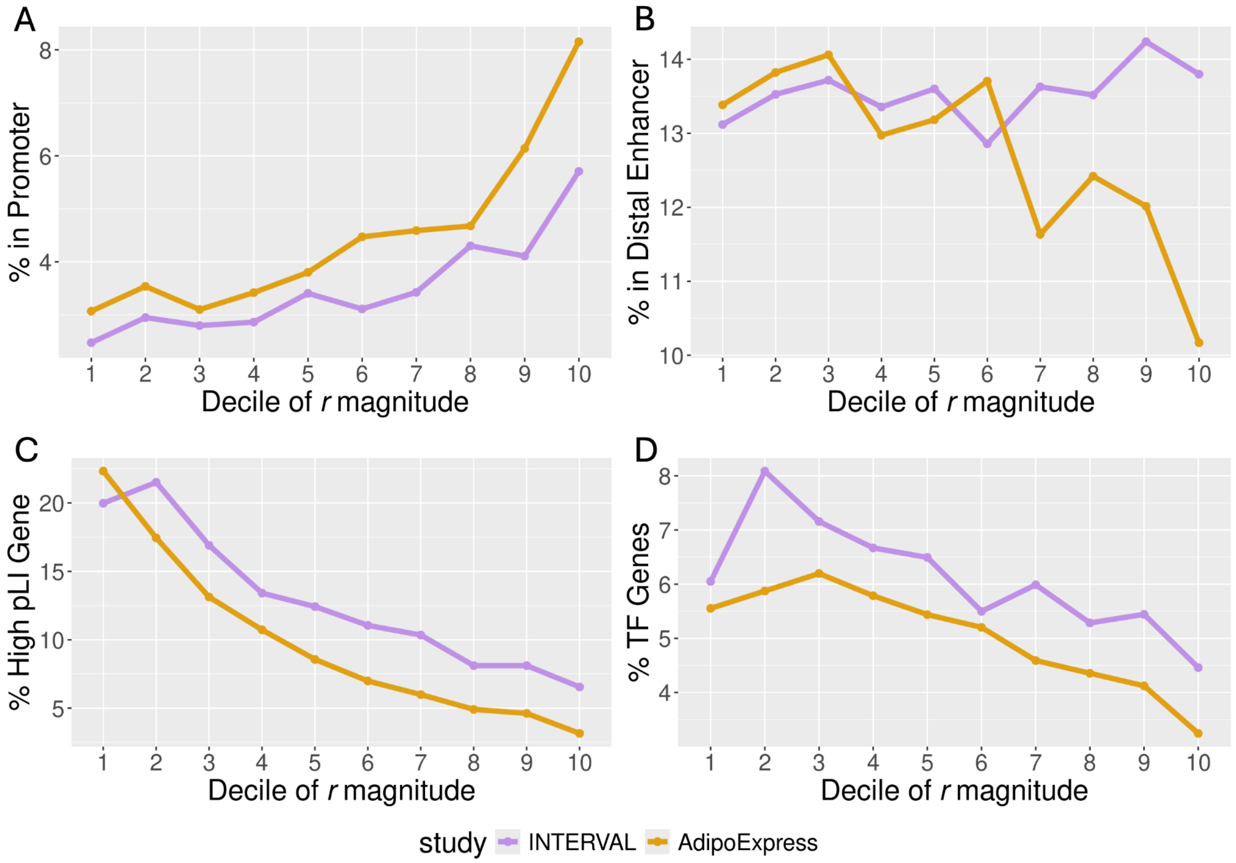

Figure S4. Trends in characteristics of eQTL signals by signal strength. Shown over the decile of r magnitude (x-axis, increasing magnitude moving from left to right) are the following: (A) percentage of signals per decile located within an ENCODE annotated promoter region or (B) distal enhancer region and (C) percentage of eGenes associated with the eQTL signal that are classified as high pLI genes or (D) transcription factors.

bioRxiv preprint doi: https://doi.org/10.1101/2025.08.05.668745; this version posted August 5, 2025. The copyright holder for this preprint (which was not certified by peer review) is the author/funder, who has granted bioRxiv a license to display the preprint in perpetuity. It is made available under aCC-BY 4.0 International license.

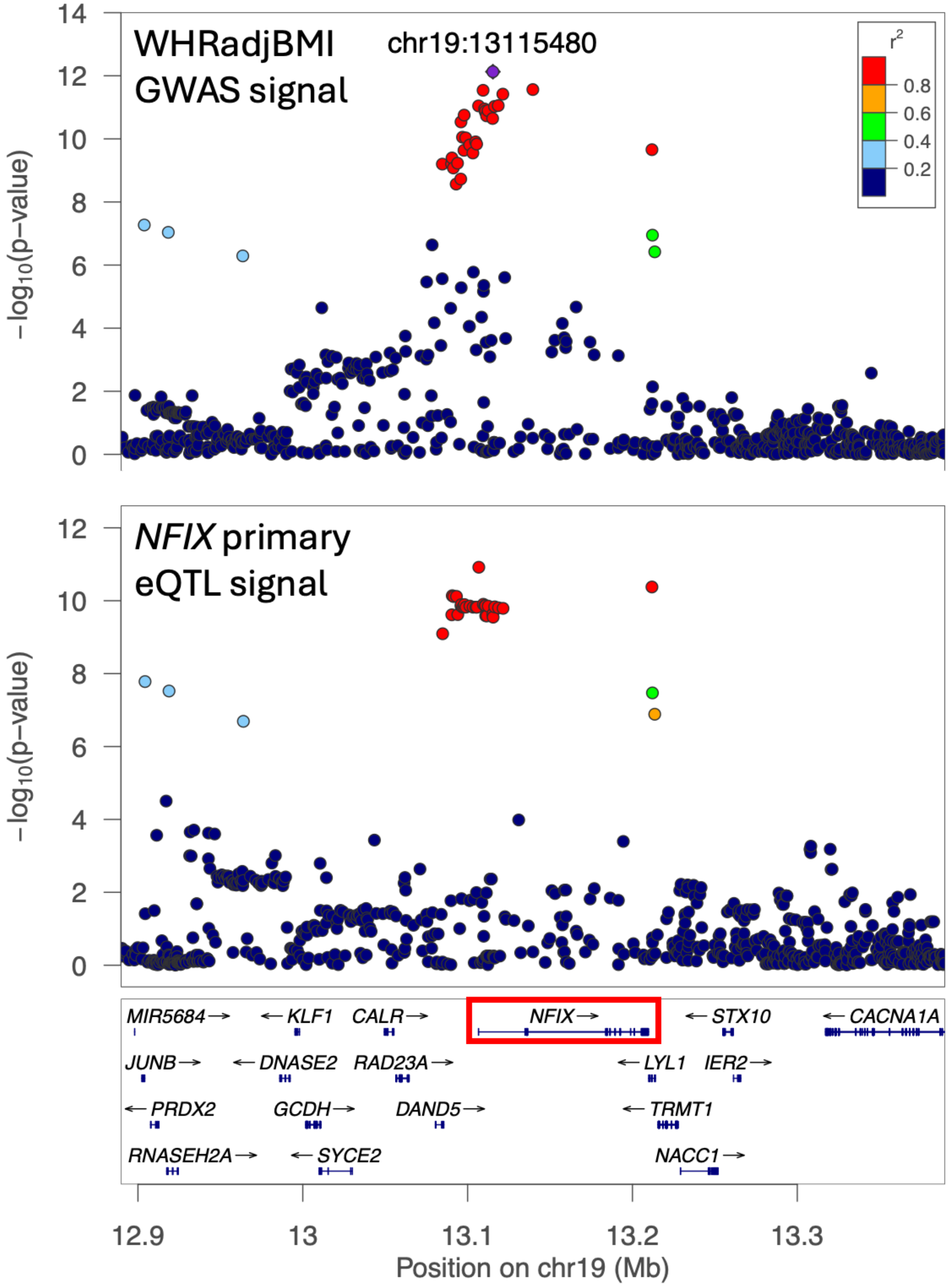

Figure S5. LocusZoom plot of colocalized GWAS-eQTL signals. Top: GWAS signal for waist-hip ratio adjusted for BMI (ref); Bottom: primary eQTL signal for *NFIX*. The eQTL signal was identified in the larger AdipoExpress study but not in METSIM-S. The lead variant for the eQTL signal has $r = 0.046$ (beta = -0.21, MAF = 0.025).

bioRxiv preprint doi: https://doi.org/10.1101/2025.08.05.668745; this version posted August 5, 2025. The copyright holder for this preprint (which was not certified by peer review) is the author/funder, who has granted bioRxiv a license to display the preprint in perpetuity. It is made available under aCC-BY 4.0 International license.

Supplemental Material and Methods

Use of Pearson correlation for computing power

Power to detect an eQTL signal is a function of both the linear coefficient describing the direction and magnitude of effect of genotype on gene expression (β) and the minor allele frequency (MAF) of the variant[44]. This dependence of power on two parameters limits comparisons of power estimates because one of the two parameters is typically held constant to observe the effect of the other on power. However, having a single metric to describe eQTL signal strength that accounts for the contribution of both β and MAF is desirable.

We assume a standard linear model

$$y = \beta_0 + x\beta_1 + \epsilon$$

where y denotes gene expression, X denotes genotype (0, 1, 2 copies of allele), and ε is an error term assumed to be normally distributed. Many eQTL analysis pipelines standardize gene expression to fit such a model by performing a rank-based inverse normal transformation on scaled RNA counts such that y is normally distributed with mean 0 and variance 1. Under Hardy-Weinberg equilibrium, the standard deviation of X can be expressed as

$$sd(X) = \sqrt{2 \cdot MAF \cdot (1 - MAF)}$$

Under this framework, the sample Pearson correlation (r) between genotype (X) and gene expression (y) can be expressed as

$$r = \hat{\beta}\sqrt{2 \cdot MAF \cdot (1 - MAF)}$$

since

bioRxiv preprint doi: https://doi.org/10.1101/2025.08.05.668745; this version posted August 5, 2025. The copyright holder for this preprint (which was not certified by peer review) is the author/funder, who has granted bioRxiv a license to display the preprint in perpetuity. It is made available under aCC-BY 4.0 International license.

$$\hat{\beta} = r\frac{sd(y)}{sd(X)}$$

and sd(y) = 1. This strategy was employed by Vosa et al[24] when estimating power in the eQTLGen study.

We use the R package powr to perform power calculations based on r. Given our ability to model $Pr(|r|)$, the distribution of the absolute value of r, we can then estimate the percentage of eQTL detected at various sample sizes using the convolution:

$$\int_0^1 \mathrm{Power}(r) Pr(|r|) d(|r|)$$

Comparing signals across GTEx and AdipoExpress

The majority of the eGenes exclusive to GTEx were not tested in AdipoExpress, so it is expected the median *r* is close to that for the common eGenes. The AdipoExpress meta-analysis required genes to exceed an expression threshold in at least two studies to be included. One potential reason for lack of expression in studies other than GTEx is the different location of the fat depot. The majority of eGenes exclusive to AdipoExpress that have significantly lower *r* values were tested in GTEx but not found to be significant.

Overlap with promoter or distal enhancer region

From the SCREEN registry of cCREs v3[45], all human cCREs (hg38) were downloaded as a single .bed file and lifted over to hg19 using the UCSC LiftOver tool. An eQTL signal was considered to overlap with a promoter region if any nucleotide of the lead variant was located within a region annotated as "PLS" or "PLS,CTCF-bound". An eQTL signal was considered to overlap with a distal enhancer region if any nucleotide of the lead variant was located within a region

bioRxiv preprint doi: https://doi.org/10.1101/2025.08.05.668745; this version posted August 5, 2025. The copyright holder for this preprint (which was not certified by peer review) is the author/funder, who has granted bioRxiv a license to display the preprint in perpetuity. It is made available under aCC-BY 4.0 International license.

annotated as “dELS” or “dELS,CTCF-bound”. Percent overlap by decile was determined by first ranking all eQTL signals by magnitude of the absolute value of r and then partitioning the entire set of eQTL signals into ten equally sized bins by rank. The number of overlapping eQTL signals divided by all eQTL signals per bin was then determined. Since the bins are equally sized, the percent overlap can be used to compare absolute counts across bins as well.

Percent high pLI or transcription factor genes

The file “gnomad.v2.1.1.lof_metrics.by_gene.txt.bgz” was downloaded from the Broad Institute website (https://gnomad.broadinstitute.org/downloads) and Ensembl IDs for genes with pLI scores ≥ 0.9 were extracted into a list of high pLI genes. eQTL signals were linked to their associated gene, which was cross-referenced with the list of high pLI genes to determine percentages per decile. The TF gene overlap was determined analogously after extracting Ensembl ID from the corresponding list.

Colocalization

For each trait, we used PLINK (v.1.90b3) to calculate the LD r^2 between all conditionally distinct GWAS lead variants and conditionally distinct adipose eQTL lead variants within 500 kb of each other using 40,000 unrelated UK Biobank (UKBB) participants as the LD reference panel[46]. If the LD r^2 was ≥0.5, we tested GWAS–eQTL pairs for colocalization using coloc (v.5.1.0.1, coloc.abf, default settings). We considered GWAS–eQTL signal pairs colocalized if the coloc PP4 was ≥0.5. For the METSIM-S comparisons, a separate colocalization analysis was not performed. Rather, we computed the LD (PLINK v.1.90b3) between all pairwise combinations of eQTLs in the smaller study with those in the meta-analysis for each eGene. A GWAS signal was considered colocalized with a METSIM-S eQTL if LD r^2 was ≥0.5 with a colocalized meta-analysis eQTL for the same gene.
